# Compound Dislocation of the Ankle and Other Injuries: Illustrating the Antiseptic System of Treatment

**Published:** 1870-09

**Authors:** Joseph Lister


### Compound Dislocation of the Ankle and Other Injuries: Illustrating the Antiseptic System of Treatment.

*By Joseph Lister, F.R.S.*

This is a paper of considerable length, contributed to the July number of the *Lancet;* it is not merely a discussion of a single case, but contains likewise the opinions of the author on certain questions pertaining to inflammation, and hence we abridge the article for our columns.

A laborer, 33 years of age, was severely injured at 6 A.M., by a railway train. When he was seen at 8.30 A.M., he was suffering considerably from shock, and, on examination, it was found that his left foot was much displaced inwards, the end of the fibula protruding through a vertical wound in the integument, two or three inches in length, the end of the protruding part being comminuted. The tip of the malleolus had been broken off, and remained attached to the external lateral ligament. The internal malleolus was of course fractured, as a necessary condition of such a displacement of the foot.

Such an injury is a most formidable one ; formerly, recoveries from it were exceptional, and surgeons came to regard amputation as the best treatment for it, in most cases.

For the purpose of facilitating the return of the protruded end of the bone, a portion of it was nipped off with cutting pliers, and, with the same object, the lower end of the rent in the skin was slightly enlarged. But, to all intents and purposes, the dislocation was simply reduced.

Watery solution of carbolic acid, as strong as it could be made (1 to 20), was thrown into the joint with a syringe, the edges of the skin being held together to prevent its escape and cause it to penetrate to all parts of the wound ; this was further promoted by manipulation of the parts, while the fluid was still in the interior. There was a time when it would have been thought dangerous to introduce so irritating a liquid into the ankle-joint ; but the transient irritation of the antiseptic is nothing compared with the far more acrid products of putrefaction. A lotion of half the strength (1 to 40) is sufficient to destroy the putrefaction of organisms in a wound just made, and made by the surgeon himself. But with an injury received sometime before the patient is seen, and in a rude way, where foreign materials with clots of blood may be lying in parts of the wound, it is proper to use as strong a solution as water will produce.

The liquid introduced having been squeezed out, a second injection was made for greater security ; the skin in the vicinity was washed with the solution to destroy organisms adhering to it or to the hairs, and an external dressing was applied. Lac plaster, containing carbolic acid, was wrapped in two layers around the limb, extending well up the leg, and embracing the heel and instep, the foot being held in good position. A cloth to absorb the serum, which would be discharged from beneath the margins of the plaster, was then bandaged on, and a splint applied to the inner aspect of the limb.

On examining the head there were found four scalp wounds, varying from two to five inches in length, three of them exposing the bone. The region was shaved and thoroughly washed with the strong antiseptic lotion ; the wounds were treated exactly like that of the ankle, except that their edges were approximated by antiseptic sutures. These were made by steeping silk for a time in a mixture of melted bee's-wax and a tenth part of carbolic acid, and being drawn through a dry cloth when removed.

The patient also had a compound fracture of the right olecranon. This was treated in the same manner as the wound of the ankle.

The dressings were changed entirely on the day after the accident. In doing this great care is requisite. The antiseptic first injected having been absorbed, the extravasated blood and dead tissue are as susceptible of putrefaction as though no such treatment had been pursued; if a single drop of serum is pressed out and then regurgitates into the interior, after being exposed even for a second to the septic influence of the air, putrefaction would be pretty certain to occur.

To guard against this risk, for the first few days, a syringe should be used, the nozzle of which is inserted beneath the margin of the lac plaster, and, as this is raised, a stream of a weak solution of carbolic acid is thrown upon the wound, until a cloth wet with the same solution can be placed over it.

After the first day, before applying the lac plaster, the wound was covered with a material to protect it from the irritating and stimulating influence of the carbolic acid in the antiseptic stratum. After the first dressing, the object which I aim at is to have the material in contact with the exposed tissue approximate as closely as possible to the bland and neutral character of the living textures. The injured tissues do not need to be *stimulated*, they need to be let alone; nature will then take care of them.

Now, of all injurious external agencies the worst is putrefaction; this we endeavor to preclude. But a substance that will destroy the life of putrefactive organisms, cannot fail to be abnormally stimulating to the exposed tissues; these must be protected from its action.

Our "protective," then, should be a material, unstimulating in itself, and impervious to carbolic acid; it must be insoluble in the discharges, and so supple that it will apply itself readily to the part. The best is made of cotton cloth, coated on one side with caoutchouc, gilded on the caoutchouc side, and then covered with a film of india-rubber applied in solution.

A very good protective is made of oiled silk, which should be brushed over with a mixture of one part dextrine, two of powdered starch, and sixteen parts of cold, watery solution of carbolic acid (1 to 20). This coating enables its surface, unlike the simple oiled silk, to become uniformly moistened, this being necessary to be done —with an antiseptic solution—when it is applied.

On the day after the accident, the cloths, plaster, and splint were found soaked with bloody discharge. On the second day, when the dressings were again changed, the cloths presented only a stain, corresponding with a few drachms of tinged serum, and the original coagulum was on a level with the surrounding skin. The dressings were then allowed to remain two days; at this time the discharge had caused only a serous stain of a few drops. This state of things resulted not merely from the antiseptic treatment, but it showed that the protective answered well its purpose. Had the antiseptic been acting directly on the wound the discharge would have been much greater, and we should have had a hollow sore, with commencing suppuration.

From the remarks of some, it would be imagined that "I regard putrefaction as the sole cause of suppuration; whereas, my treatment of abscess depends upon the fact that pus, in the unopened cavity, being the result of inflammatory stimulus, without atmospheric influence, is free from putrefaction; so it is needless to apply the antiseptic to the interior, all that is requisite being to provide exit for the discharge, while guarding against the entrance of putrefactive fermentation."

Carbolic acid freely applied to a wound, instead of preventing suppuration, will itself, being a stimulating substance, induce suppuration, by long continued action on the tissues.

Facts observed in the antiseptic system of treatment have thrown great light on the causes of suppuration; it may be well to state here the "conclusions to which I have been led."

The tissues of the living body are liable to a temporary impairment or suspension of vital energy as the result of extreme irritation; this condition is the essence of *intense* inflammation, and may be induced in two ways, *viz.:* either by the direct operation of a noxious agent on the tissues, or indirectly through the medium of the nervous system. The same law appears to hold with regard to the causes of the exaggerated but feeble cell-development resulting from the continued action of some abnormal stimulus in a less intense form, giving rise to the various phenomena of inflammatory hypertrophy, granulation, and suppuration; the pus cells being the extreme of excess of quantity, and impairment of quality in the products of abnormally excited nutrition. Thus there are two groups of causes of suppuration: first, those that operate through the nervous system, or the inflammatory class, of which abscess is a typical example; and, secondly, noxious agents or stimuli acting directly on tissues. The latter group are stimulating salts or chemical stimuli. These are best studied in the behavior of a healing ulcer under different kinds of treatment. Small granulating sores sometimes heal by scabbing; when the surface is thus protected by a crust from external influences, there is no further effusion of pus or serum. This proves that granulations have no inherent tendency to form pus, but only do it when stimulated. Two granulating surfaces placed in contact with each other will coalesce; this would be impossible if they continued to suppurate; and their juxtaposition could oppose no obstacle to the pus formation, if they had any innate disposition to it. But their contact excludes the operation of external agents upon them, they cease to discharge, and unite. The wall of an abscess is similar in nature to the granulations of a sore, and is often regarded as essentially "pyogenic," but if the abscess be opened antiseptically, the pyogenic membrane, relieved from the inflammatory stimulus which the tension of the pus induced, and being protected from the access of the stimulus of putrefaction, is left free from all disturbance, and never forms another drop of pus.

So far from granulations having any inherent tendency to form pus corpuscles, they are ever disposed to develop into higher forms as soon as left free from preternatural excitement. In the healing ulcer, the granulations covered by the newly-formed pellicle of epidermis at the edge of the sore, are as truly granulations as when exposed, but they are no sooner protected from external stimulus than they proceed to develop into the more and more perfect fibrous tissue of the cicatrix.

When carbolic acid or chloride of zinc is applied, properly diluted, to a healthy granulating sore, no redness of the surrounding skin, or other inflammatory disturbance is produced; yet the granulations exposed to the liquid are excited to superficial suppuration, while those protected from it by the pellicle of epidermis form no pus. Here, then, we have absence of inflammatory stimulus, but the chemical stimulus urges the granulation to develop pus

If the sore is treated with water dressings, the serum first exuded putrefies in the lint, and the products being acrid salts stimulate the granulations to the formation of pus. Thus, in their effect on a granulating sore, an antiseptic and a putrid dressing are alike; both excite suppuration by stimulating the granulations.

But, in their operation on a recent wound, there is this difference between them, that the antiseptic stimulates only the surface to which it is applied, and the discharge which it induces dilutes it and renders it less stimulating; but putrefaction being a fermentation, the ferment spreads to all the recesses of the wound, wherever blood, serum, or dead matter afford nidus and pabulum for its development.

Antiseptics, then, though they produce suppuration when applied to a recent wound, are superficial in their action and trivial, compared with the effect of putrefaction.

Speaking of the condition of *time* in suppuration, he says that it is only when tissue has been gradually degraded by abnormal stimulation to a state of granulation that pus is formed. To accomplish this in a recent wound in healthy tissue requires three or four days when subjected to the action of putrefying material.

The same holds with regard to the inflammatory stimulus; it does not induce suppuration in a day, but requires time.

The case under consideration progressed rapidly and favorably, all the wounds healing with the formation of hardly a particle of pus. The oiled silk protective having been used in two, and sometimes three layers, the results approached closely those which are theoretically attainable. Some of the smaller portions of the slough were entirely removed by absorption, and their place filled by vascular new tissue. Five weeks after the accident, the process of organization and vascularization [in the ankle] had made great progress, and the large mass of dead tissue, though superficially situated, being protected from the disturbing influences of external agency, was undergoing the same kind of change as is experienced by parts deprived of vitality in the sub cutaneous injury of simple fracture. At this period, the original clot was still to be seen on a level with the surrounding skin, but diminished by contraction and cicatrization. An open sore healing by cicatrization, without either suppuration or granulation, is something new in the history of surgery, though just what we might expect from our knowledge of healing by scabbing.

